# The Effect of Bromine and Iodine on the Plant Growth, Phytochemical Composition and Antioxidant Capacity of Dandelion (*Taraxacum officinale* F.H. Wiggers Coll.) Plants

**DOI:** 10.3390/molecules30102239

**Published:** 2025-05-21

**Authors:** Iwona Ledwożyw-Smoleń, Sylwester Smoleń, Marta Liszka-Skoczylas, Joanna Pitala, Łukasz Skoczylas

**Affiliations:** 1 Department of Plant Biology and Biotechnology, Faculty of Biotechnology and Horticulture, University of Agriculture in Krakow, al. Mickiewicza 21, 31-120 Krakow, Poland; sylwester.smolen@urk.edu.pl; 2 Department of Engineering and Machinery for Food Industry, Faculty of Food Technology, University of Agriculture in Krakow, al. Mickiewicza 21, 31-120 Krakow, Poland; marta.liszka-skoczylas@urk.edu.pl; 3 Laboratory of Mass Spectrometry, Faculty of Biotechnology and Horticulture, University of Agriculture in Krakow, al. Mickiewicza 21, 31-120 Krakow, Poland; joanna.m.pitala@gmail.com; 4 Department of Plant Product Technology and Nutrition Hygiene, Faculty of Food Technology, University of Agriculture in Krakow, al. Mickiewicza 21, 31-120 Krakow, Poland; lukasz.skoczylas@urk.edu.pl

**Keywords:** iodine, bromine, biofortification, chemical composition, dandelion, antioxidant capacity

## Abstract

Iodine is a crucial microelement for humans, and iodine deficiencies may be reduced through the consumption of iodine-enriched plants. The possible effects of exogenous bromine regarding plant growth, iodine biofortification efficiency, and the chemical composition of cultivated plants have not been previously evaluated. A two-year pot cultivation of dandelion was conducted, applying KBr and KIO_3_ in the following combinations: (1) Control, (2) 10 µM I, (3) 50 µM I, (4) 10 µM Br, (5) 50 µM Br, (6) 10 µM I + 10 µM Br, and (7) 50 µM I + 50 µM Br. An increased plant biomass indicated the low toxicity of the tested doses of I and Br for dandelion. However, a slightly increased antioxidant capacity in the leaves and roots and higher proline content in the leaves may suggest a potential stress effect of iodine and/or bromine accumulation for plants. The Br:I ratios observed in biofortified leaves and roots indicate the need to monitor bromine levels in soils or substrates used for plant cultivation in order to reduce the risk of excessive Br contents in iodine-enriched plants.

## 1. Introduction

### 1.1. Iodine and Bromine Biogeochemistry

Iodine (I) and bromine (Br) belong to group 17 of the elements (the halogens); in the natural environment, they always occur together. Br, I, and other halogens are characterized by high chemical activity in the formed monovalent anions as related to their electronegativity values [1]. The average Br:I weight ratio on the earth’s surface is 1:0.005; in oceans, the average ratio is 1:0.008 [2]; and for rainfall and aerosols, the average ratios are 1:0.20 and 1:0.14, respectively [3]. The content of Br and I in the land environment varies depending on the soil type as well as the distance from seas and oceans—the main reservoirs of these elements [4]. The Br:I weight ratio in peat soils depends on the soil’s origin and is within the range of 35.0–2.9:1 [5]. Organic forms of I and Br (mostly bound to humic and fulvic acids) seem to be the dominant forms of these elements in peat and organic soils, while inorganic bromides and iodides/iodates contribute a much lesser share of the total [5,6].

Iodine is a crucial element for the proper functioning of human and animal organisms. It is present in the structure of the hormones produced by the thyroid gland, namely triiodothyronine (T3) and thyroxine (T4), and plays an important role in the development of neural cells and the brain during the prenatal stage [7]. In plants, iodine is considered a micronutrient [8] or a beneficial element [7]; it was demonstrated that low doses of that element positively affect plant growth, flowering, and antioxidant capacity, as well as the effects under stress conditions [9]. In comparison to iodine, Br is a relatively less known element. In the older literature, bromine was classified as a non-essential element for animals or plants [4]. Numerous researchers have noted that the physiological and biochemical role of Br in plants, animals, and human organisms has not yet been determined [1,10]. However, according to biogenic element classification, bromine is an important element for living organisms [1]. It should be mentioned that an excess of both iodine [7,11] and bromine [1] is harmful, or even toxic, for plants, animals, and human organisms.

The harmful effects of Br on human and animal organisms are relatively well documented due to the need to evaluate the toxicity of Br in regions with an increased level of its accumulation [1]. The main anthropogenic sources of bromine in the environment include fossil fuel combustion, soil fumigation with methyl bromide and dibromoethane as a method of nematode control, and the application of brominated flame retardants in the plastic industry [12]. A relation between thyroid pathologies and a high concentration of bromine in the environment has been confirmed [13]. In mammalian organisms, Br has a tendency to replace I, which leads to a decrease in the I level in thyroid and mammary glands, as well as in skin [14]. On the other hand, a limited number of bromine compounds are of pharmaceutical use as antibronchitis [15] or antispasmodic agents [16]. There are no critical limits for Br levels in soils or food; however, the recommended daily intake of bromide ions was set as up to 1 mg/kg of body weight/day [17].

The physiological, biochemical, and molecular responses of higher plants towards Br have not yet been sufficiently described in the literature. It has been reported that bromide ions may be involved in reducing the phytotoxicity of selected organic compounds, such as sulfonamide antibiotics, e.g., sulfadiazine [18], or phenolic pollutants [19], which may be of substantial importance for phytoremediation purposes. However, excessive Br leads to low germination rates, chlorosis and necrosis of leaf edges [20], and Cl displacement, which increases the permeability of cell membranes, among other impacts. A similar impact is observed to result from excessive I content in plants and is directly related to the chemical properties of Br and I [11]. The natural sources of Br and I for plants include soils, dry and wet deposition, and mineral and organic fertilizers. The content of I and Br in the soil is directly related to its organic matter [4]. Among the mineral fertilizers, the highest contents of Br are noted in potassium chlorides, while the highest contents of I are observed in Chilean sodium nitrate [21]. As a strong oxidant agent, Br can oxidize nitrites to nitrates, sulfur and sulfites to sulfuric acid, ammonia to nitrogen, and iodides to free iodine; these processes take place in aqueous environments, including soil solutions [1]. Therefore, an increased concentration of Br in the rhizosphere may substantially affect the availability and uptake of mineral nutrients by plants. This may be of substantial importance, particularly regarding the possibility of increasing the content of mineral nutrients in crop yields and therefore improving human nutrition through essential microelements such as iron, zinc, selenium, and iodine [22].

The iodine biofortification of plants has been proposed as a method of improving the iodine status of consumers [23]. Numerous studies have been conducted on the application of iodine to forage and food crops, including medicinal plant species, such as basil [7,24], with the use of various cultivation methods: pot experiments, field studies, and soilless and hydroponic conditions. The level of uptake and accumulation of inorganic iodine forms (KI, KIO_3_, and NaI) has mainly been determined alongside its effect on selected biochemical and physiological parameters of plants [7]. Recently, some works have evaluated the potential for increasing iodine content in tomatoes [25], sweetcorn [26], and lettuce [27] using organic sources such as iodosalicylates and iodobenzoates.

Given the wide range of natural bromine content in soils, it is important to evaluate the extent to which this element affects the uptake and accumulation of iodine by plants. For this reason, analytical protocols are needed to determine the total content of both Br and I [28,29,30]. Evaluating the uptake of Br and I by higher plants is particularly important for characterizing its transfer within a soil/water-plants/animals-human food chain, especially when considering its interaction [1]. Furthermore, exogenous Br and I most likely induce specific physiological and biochemical responses in plants, which can be manifested by the accumulation of various plant metabolites such as phytohormones and phenolic compounds, as well as by the modified antioxidant capacity in plant extracts.

### 1.2. Dandelion Plants—Medicinal Properties, Endogenous Content of Br and I, Iodine Biofortification of Plants

The plants of common dandelion (*Taraxacum officinale* F.H. Wiggers coll.) are commonly used as pharmaceutical raw materials. The leaves of dandelion are characterized by their immunoprotective, anti-colitis, hepatoprotective, diuretic, anti-diabetic, anti-obesity, anti-arthritic, antibacterial, antifungal, antiviral, antioxidant, and anti-cancer properties [31]. These beneficial effects are related to the presence of various metabolites, such as phenolic acids (e.g., chlorogenic acid, caffeic acid, and chicoric acid); glycosides (e.g., esculin); sesquiterpene lactones (e.g., ixerin D, taraxacolide-O-β-glucopyranoside, taraxacoside, and taraxinic acid); triterpenes (e.g., α-amyrin); sterols (e.g., β-sitosterol, stigmasterol, and taraxasterol); polysaccharides (e.g., inulin); flavonoids (e.g., chrysoeriol, luteolin-7-glucoside, and quercetin); and carotenoids [32,33]. Dandelion plants can also be used as bioindicators of environmental pollution [34,35], as they can accumulate substantial amounts of various trace elements from contaminated soils and wet and dry deposits without showing any visible toxic symptoms, even at relatively wide levels [36].

Hartmans [37] conducted one of the first studies to document the endogenous iodine content of dandelion leaves and its subsequent changes after the application of KI to the soil. However, the level of iodine in dandelion roots was not determined in that work. Information on the endogenous bromine content in dandelion plants is scarce. Baidoo et al. [38] determined the concentration of Br in the leaves of the plants of this species grown in Ghana to be on the level of 10.32 mg·kg^−1^. No data on the Br:I ratio in dandelion leaves and plants has been found—such information would be particularly important for developing agrotechnical recommendations for iodine biofortification of this species. Increasing the iodine content of dandelion plants, being a herbal species, may be considered a method of improving their health-promoting properties. Given its widespread occurrence in various climatic zones and its high tolerance to increased accumulation of various elements, dandelion seems an appropriate species for monitoring the possible interactions between iodine and bromine.

The aim of this study was to evaluate, for the first time, the effect of the simultaneous application of potassium bromide (KBr) and potassium iodate (KIO_3_) to dandelion plants. The iodate form of iodine was chosen based on its applicability for biofortification purposes and its lower toxicity to plants as compared to iodide forms [8,39]. The study aimed to determine the extent to which exogenous bromine would affect the yield, the efficiency of iodine accumulation, and the content of selected bioactive compounds, phytohormones, as well as the antioxidant capacity of dandelion plants. Furthermore, Br accumulation in plant leaves and roots was planned to be monitored alongside the changes in the Br:I ratio in the respective plant parts.

## 2. Results

### 2.1. Biomass and Dry Weight in Roots and Leaves

The tested combinations had no influence on the average leaf biomass from a single plant or dry matter content in roots and leaves (Table 1). Compared to the control combination, iodine and bromine had a positive effect on the biomass of dandelion roots and whole plants, as did the simultaneous application of both elements (in both doses). Increasing the dose of both tested elements (applied separately or simultaneously) from 10 µM to 50 µM did not significantly change the biomass of roots and whole plants. The most favorable effect on root and plant biomass was observed with the simultaneous application of I + Br (Table 1). Importantly, no visual symptoms of I and/or Br toxicity were observed.

### 2.2. Content of Iodine and Bromine and Its Quantitative Ratio in Roots and Leaves

The iodine content of dandelion plants remained similar in the control combination, and after bromine application in both doses remained at a similar level, i.e., 0.34, 0.44, and 0.27 mg I·kg^−1^ DW in the leaves versus 0.29, 0.35, and 0.33 mg I·kg^−1^ DW in the roots, respectively (Table 2). On the other hand, the bromine content of the leaves of the control plants and those after iodine application in both doses (9.04, 8.36, and 11.08 mg Br·kg^−1^ DW, respectively) was approximately twice that in the roots from the respective combinations (4.06, 4.38, and 5.40 mg Br·kg^−1^ DW, Table 2).

Introduction of iodine solutions, either separately or together with bromine, caused a significant and dose-dependent increase in iodine content in dandelion plants, and leaves contained 3.6 to 5.9 times more of that element than roots (Table 2). Applying a higher dose of both I and Br together (50 µM I + 50 µM Br) resulted in greater iodine accumulation in dandelion leaves compared to applying 50 µM I alone. On the other hand, the additional application of bromine together with 50 µM I decreased the level of iodine in dandelion roots compared to the combination with iodine applied alone.

The bromine content in dandelion leaves and roots increased with the dose of Br applied either alone or together with iodine (Table 2); additional application of iodine increased the bromine content only in the leaves. The bromine content in the leaves of plants treated with bromine alone was 2.7 and 3.8 times higher than in the roots (for 10 µM and 50 µM Br, respectively). The bromine content in the leaves from the I + Br combination was 4.3 and 6.3 times higher than in the roots for the 10 and 50 µM doses, respectively.

Importantly, despite the same doses of both elements being applied, the accumulation of bromine in dandelion leaves and roots was much higher than that of iodine. When applied together, the level of Br in the leaves was 13.8 and 7.9 times higher than the level of I, while the roots contained 11.8 and 7.5 times more bromine than iodine (for 10 and 50 µM I + Br doses, respectively).

The Br:I ratio in the control plants was estimated at 26.6:1 and 14.0:1 levels for leaves and roots, respectively. Applying iodine alone in both tested doses significantly limited the Br:I ratio to the level of 0.4:1 in leaves and 0.9:1 in roots from the 50 µM I dose. On the other hand, the application of a 50 µM Br dose significantly increased the values of the Br:I ratio to the level of 579.5:1 in leaves and 125.5:1 in roots. Simultaneous application of I and Br led to 2–3 times lower Br:I ratios in both leaves and roots than in the control combination.

### 2.3. Content of Salicylic, Benzoic, 5-Iodosalicylic, and 5-Bromosalicylic Acids and Iodotyrosine

**Figure 1:**
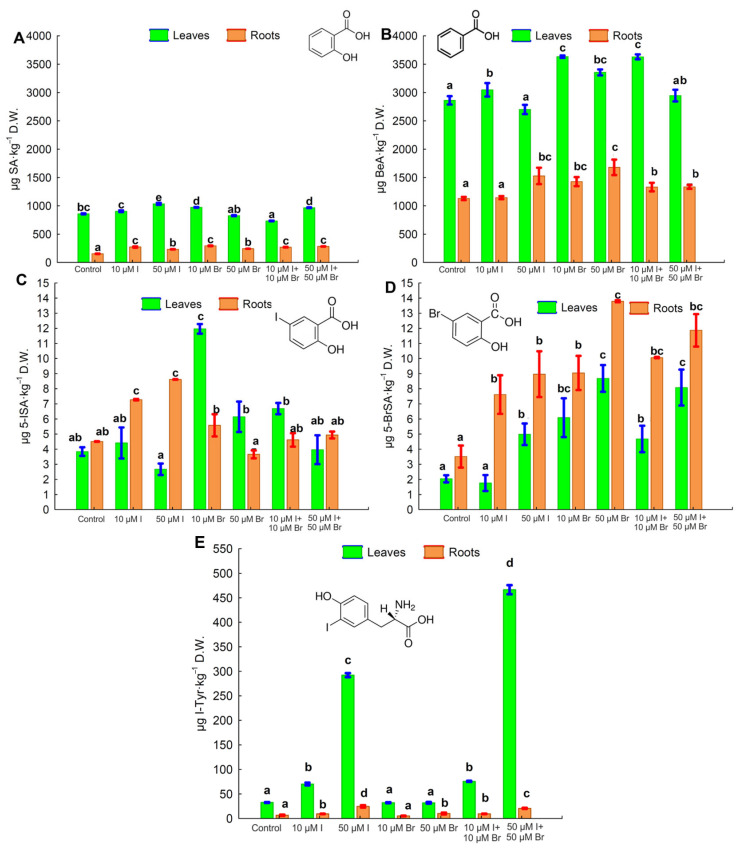
Concentrations of salicylic acid (SA) (**A**), benzoic acid (BeA) (**B**), 5-iodosalicylic acid (5-ISA) (**C**), 5-bromosalicylic acid (5-BrSA), (**D**) and iodotyrosine (I-Tyr) (**E**) in dandelion leaves and roots. Means followed by different letters for treatments, separately for leaves and roots, differ significantly at *p* < 0.05. Bars indicate standard deviation (means from one cycle of dandelion plant cultivation; n = 4).

Most of the tested I and Br applications resulted in the increased accumulation of salicylic acid (SA) and benzoic acid (BeA) in dandelion plants (Figure 1A,B). Leaves from all combinations contained higher levels of SA and BeA than roots. The application of a higher iodine dose (50 µM I, either alone or together with bromine) significantly increased the content of SA and decreased the level of BeA in the leaves compared to the respective 10 µM I combinations. Similarly, application of 50 µM Br decreased the level of BeA in the leaves as compared to the 10 µM Br dose.

The application of KIO_3_ without KBr at 10 and 50 µM I doses resulted in obtaining the highest 5-ISA content in roots (Figure 1C). In comparison to these treatments, the combined application of I + Br decreased the 5-ISA content. The highest 5-ISA content in the leaves was found after the application of 10 µM Br, which also increased the 5-ISA content in roots compared to the control. The applications of 50 µM Br and 50 µM I + Br caused the greatest increase in the 5-BrSA content in roots and leaves as compared to the control (Figure 1D). The lower dose of Br (10 µM applied alone or together with iodine) had a smaller effect on increasing the 5-BrSA content in roots and leaves. Applying iodine alone in two doses of 10 µM I and 50 µM I caused a simultaneous increase in the content of 5-ISA and 5-BrSA in roots as compared to the control (Figure 1C,D).

The application of iodine and bromine significantly affected the I-Tyr content in dandelion leaves and roots (Figure 1E). The leaves of plants from all tested combinations contained more I-Tyr than roots. Applying a higher iodine dose (50 µM I, either alone or in combination with bromine) significantly increased the content of I-Tyr in the leaves and roots of dandelion as compared to respective 10 µM I combinations (Figure 1B,C).

### 2.4. Content of Esculin, Chlorogenic Acid, and Total Phenolic Compounds

Compared to the control combination, the esculin content in dandelion leaves and roots was unaffected by the applied I and Br compounds. The highest leaf content values of chlorogenic acid and total phenolic compounds were observed following the application of 50 µM I (Table 3). A simultaneous increase in the root content of chlorogenic acid and phenolic compounds was only noted for 10 µM Br. The second highest level of chlorogenic acid in the roots was noted in the plants from the 50 µM I treatment, while the total phenolic compounds—after the application of 50 µM Br.

### 2.5. Antioxidant Capacity of Dandelion Plants

Significant effects of I and Br application were noted with respect to the antioxidant capacity of dandelion leaf and root extracts, as measured by the CUPRAC, FRAP, and DPPH methods (Table 4). Some of the highest values of CUPRAC and DPPH antioxidant capacity were observed in the leaves of plants from the 50 µM I combination. On the other hand, the highest CUPRAC and FRAP values were noted in the leaves of plants grown in the presence of 10 µM I + 10 µM Br. In the case of dandelion roots, the highest antioxidant capacity as measured by the three methods (CUPRAC, FRAP, and DPPH) was noted in the plants from the 10 µM Br combination.

Increasing the iodine dose from 10 to 50 µM I, when applied alone, appeared to increase the antioxidant capacity of leaf and root extracts, with the exception of the DPPH values in dandelion leaves (Table 4). On the other hand, increasing the bromine dose to 50 µM Br reduced the antioxidant capacity of dandelion roots when that element was applied alone (without iodine). The dose of iodine and bromine introduced together had no significant effect on the antioxidant capacity of dandelion leaves and roots.

### 2.6. Concentrations of Proline as Well as Phytohormones: IAA, GA_3_, GA_4_, JA, and ABA

Compared to the control, all tested treatments with the application of iodine and bromine increased the proline content in the leaves as well as the level of IAA and JA in the roots of dandelion (Table 5). A decrease in JA content in the leaves was also observed. No effect of the tested compounds was noted regarding the level of GA_4_ in the leaves and roots or the content of GA_3_ in the roots of dandelion.

The greatest decrease in the IAA content in leaves was observed after the application of 50 µM I + Br (Table 5). The lowest concentration of JA in the leaves was noted in the combinations with 50 µM I and 50 µM Br applied independently. Fertigation of plants with 50 µM I led to the greatest decrease in proline content as well as a significant increase in JA content in dandelion roots.

Plants fertigated with the 10 µM I + Br solution were characterized by the highest contents of GA_3_ in the leaves as well as the greatest accumulation of IAA and JA in the roots as compared to other tested treatments, including the control (Table 5). The lowest level of GA_3_ in the leaves was noted in the plants from the 10 µM Br application.

The lowest accumulation of ABA was found in the roots of plants fertigated with the 50 µM I + Br solution as well as in the leaves of plants from the 10 µM I + Br treatment. The highest leaf content of ABA (which also exceeded the control value) was found in plants fertigated with 10 µM I.

## 3. Discussion

### 3.1. Accumulation of I and Br in Dandelion Leaves and Roots

The mineral composition of dandelion plants reflects the bioavailability of individual chemical elements, i.e., mineral nutrients and trace elements in the environment [36,40]. Endogenous iodine content in dandelion plants depends on the soil type and its physicochemical characteristics, including the soil level of iodine, and was previously estimated within a range from 0.10 mg I·kg^−1^ DW to 2.56 mg I·kg^−1^ DW [37]. In the current study (Figure 1), iodine content in the leaves and roots of the control plants was determined as 0.34 and 0.29 mg I·kg^−1^ DW, respectively. This is similar to the lower value in the range presented by Hartmans et al. [37].

As with other crops, an increased accumulation of Br in dandelion leaves is strictly related to the anthropogenic contamination of soils [40]. The level of Br accumulation may increase differentially depending on the applied Br compound and the tested plant species. After applying 0 to 1.25 mmol·L^−1^ of NaBr and KBr during seed germination on Petri dishes, the resulting wheat seedlings contained more Br than pea seedlings. The application of KBr led to higher Br accumulation as compared to NaBr treatment [41]. Similar studies conducted in soil revealed that wheat seedlings were more tolerant to Br as compared to pea plants [42]. Another factor affecting the level of bromide accumulation in higher plants is the method of plant cultivation. Shtangeeva et al. [43] revealed that after a one-time soil application of Br (as NdBr_3_) in a dose of 50 mg·L^−1^ (approximately 625 µM Br), the content of Br in leaves and roots of wheat seedlings was 28.6 and 31.0 mg Br·kg^−1^ DW, respectively. Plants from hydroponic cultivation accumulated substantially higher amounts of this element, namely 6280 (leaves) and 3351 mg Br·kg^−1^ DW (roots). The highest noted level of Br noted in dandelion plants in the present study was 241 mg Br·kg^−1^ DW (50 µM I + 50 µM Br treatment), which was comparable to the values noted in plants fertilized with potassium fertilizers characterized by high Br contents [44].

The accumulation of Br in dandelion leaves and roots was substantially higher than that of I. In the case of simultaneous application of both elements, up to 13.8 times more Br than I was found in leaves. Studies conducted by Whiskerman [3] also revealed that *Lolium multiflorum* plants took up more Br than I. These results differ from those obtained for rice, for which iodine accumulation in the roots was up to 15 times higher than bromine accumulation, while the respective values in the shoots remained similar. This observation has been explained based on the ability of rice roots to oxidize iodide but not bromide ions, which in turn may result in increased and preferential uptake of obtained molecular iodine by rice plants [45].

The calculated Br:I ratio in the control dandelion plants was 26.5:1 and 14.0:1 for leaves and roots, respectively. These values were closer to the Br:I ratio noted in the water used for watering the plants (27.9:1) than in the substrate before plant cultivation (2.2:1). After the sole application of 50 µM bromine, the Br:I ratio increased to the maximum values of 579.5:1 and 125.5:1 observed for dandelion leaves and roots, respectively. As no direct information on the Br:I ratio in plants could be found, we attempted to estimate this characteristic based on the results presented in various studies. For soybean plants, the values of the Br:I ratio depended on soil type, varying from 46.1:1 for sandy Regosols to 174.1:1 for Andosols [4]. In other studies, the endogenous Br:I ratio in the roots and leaves of control wheat seedlings grown in the soil was 22:1 and 124:1, respectively. For hydroponic cultivation these values were 20:1 and 574:1, respectively [43]. Data presented by Nascimento et al. [29] reveal that the calculated Br:I ratio varied from 4:1 (*Illicium verum* Hook.f.) to 1093:1 (*Trigonella foenum-graecum* L.) in eight species of locally grown Brazilian medicinal plants. Studies conducted by Tagami et al. [28] on plants cultivated in the area of Aomori Prefecture (Japan) showed that a Br:I ratio was approximately 600:1 in potato, 1000:1 in radish, 100:1 in spinach and grasses, and 50:1 in komatsuna (*Brassica rapa* var. *perviridis* L. H. Bailey). In various types of plant-based certified reference materials used for validating the measurement method, the Br:I ratios were as follows: 11:1 (apple leaves, [28]) as well as 4:1 (citrus leaves) and 36:1 (peach leaves, [29]). After the application of NdBr_3_, the Br:I ratio in leaves increased to 15,232:1 and 4917:1 for hydroponic and soil cultivation, respectively [43]. Therefore, it can be concluded that the bromine-to-iodine ratio in higher plants varies significantly depending on the species, as well as on the method of cultivation, soil type, soil chemical characteristics, and the level of Br and I available to the roots.

In the current study, a stimulating effect of I and Br on the biomass of roots and whole plants was noted, particularly after the joint application of 50 µM I + 50 µM Br. Leaf biomass remained similar in all tested combinations (Table 1). The increase in plant biomass after iodine application stays in agreement with previous studies on lettuce [46] and sweet pepper seedlings [47], in which iodine was applied in relatively small doses. Recent molecular studies have revealed the potential impact of iodine on the modification of selected proteins in Arabidopsis leaves. Of particular interest in terms of plant growth is the fact that most of the modified proteins were related to photosynthesis [8]. The same studies also found that the application of 10 µM KBr significantly increased the fresh biomass of *Arabidopsis* plants, while 30 µM KBr had no effect. Other studies on bromide application have shown that the effect of these compounds on plant growth depends on the species: wheat plants grew better in the presence of low doses of bromide in the soil, while the biomass of pea plants decreased significantly. Interestingly, the type of compound applied (KBr, NaBr) did not modify the observed results [48]. However, a decrease in the biomass of oat and pea plants was noted when NH_4_Br was used [49].

### 3.2. Effect of Br and I on Phytohormone Content in Dandelion Plants

Kiferle et al. [8] revealed differing molecular responses of *Arabidopsis* plants to iodine (KI and NaI) and bromine (KBr), as determined by microarray analysis. No genes were up-regulated by bromine application, while only two genes in shoots and three genes in roots were down-regulated. On the other hand, iodine compounds were associated with the up-regulation of 33 and 398 genes in shoots and roots, as well as the down-regulation of 15 and 133 genes in the shoots and roots of *Arabidopsis* [8]. Additionally, the application of KI and NaI induced the flowering process in *Arabidopsis,* whereas such an effect was not observed after KBr treatment. This indicates a stronger effect of I than Br on physiological and molecular processes that take place in *Arabidopsis* plants [8].

The positive effects of iodine and bromine on plant biomass appear to be related to increased IAA (indole-3-acetic acid, auxin) concentrations in dandelion roots—a phytohormone responsible for stimulating root cell growth [50,51]. Studies on wheat plants revealed that low doses of bromides increased the level of IAA in the leaves under the stress of phenolic pollutants [19] or sulfonamide antibiotics [18]. On the other hand, an increase in the content of JA (jasmonic acid) was noted in the roots of dandelion from the combinations with I, Br, and I + Br, with no effect on the abscisic acid (ABA) level (with the exception of 50 µM I + Br). Jasmonic acid [52] and abscisic acid [53] are classified as the inhibitors of root growth and development. However, some studies suggest that ABA can have a positive effect on root growth, particularly when present or applied exogenously in low doses [54].

Studies on sugar corn [26] or tomato seedlings [55] confirm a positive effect of I application on the root growth and development of dandelion plants. The physiological and biochemical mechanisms of the tested applications of Br, I, and Br + I on root growth are most likely related to the interaction between individual phytohormones (IAA, GA_3_, GA_4_, JA, and ABA) and BeA and SA. It can also be hypothesized that the positive effect of iodine on the biomass of roots and whole dandelion plants results from the complex interaction between IAA, ABA, and JA. This may also be linked to the previously noted positive influence of iodine on plants, without its necessity for the completion of a full growth cycle, which is a main criterion for its classification as a beneficial element for plants [56,57].

The obtained level of I and Br accumulation in dandelion leaves (including iodine and bromine derivatives of BeA and SA) was not toxic to the plants. This conclusion is substantiated by the low increase in proline levels (plant stress indicator; [58]) in the leaves and no decrease in plant biomass after all treatments involving the application of iodine and/or bromine applications. Additionally, no general increase in the level of growth inhibitors, such as JA and ABA, was noted in leaves, further suggesting that the accumulation of iodine and bromine in the plants remained within the safe levels.

The endogenous content of gibberellins (GA_1_, GA_3_, GA_4_, GA_5_, GA_7_, GA_8_, GA_9_, and GA_13_) in dandelion plants is generally low and varies depending on the vegetation stage. The process of dandelion leaf senescence is also related to the levels of this group of phytohormones [59]. GA_4_ has been revealed to be the most active form of gibberellins in *A. thaliana*, regulating both shoot elongation and flower initiation of *A. thaliana* under short-day conditions [60]. Previous studies have demonstrated that exogenous iodine can induce the process of flowering in *A. thaliana*, which was not observed with bromine [8]. In the current study, the dandelion plants were collected at the rosette stage before the flowering initiation. Therefore, the observed changes in the content of GA_3_ (and, to a lesser extent, GA_4_ content) were related to plant response to the applied elements but did not reflect the final content of bioactive compounds such as esculin and chlorogenic acid, or dandelion growth and leaf biomass. An increase in root biomass after the application of I and/or Br may result from the increase in the content of BeA, SA, and 5-BrSA in the roots. This may be further substantiated by the previously reported increase in dandelion biomass under the influence of exogenous SA [61]. In the latter study, the correlation between flavonoid content and SA and GA_3_ levels in dandelion was also revealed [61]. In the current study, the application of I and/or Br also tended to slightly increase the content of chlorogenic acid in the roots.

### 3.3. Antioxidant Capacity of Dandelion Plants

Modifications in the functioning of the antioxidant system of plants are an indicator of the possible effects of the tested factors on the physiological and biochemical processes in plants. This is also a parameter of great importance for the consumers, as a diet rich in antioxidant compounds has a positive impact on human health. In the present study, increased accumulation of iodine and bromine was found to result in the increased antioxidant capacity of dandelion leaves and roots (Table 4). In the case of roots, the increased antioxidant capacity, as measured by all three methods, can be directly attributed to the accompanying increase in the content of phenolic compounds. Due to the lack of studies on the simultaneous application of I and Br during plant cultivation, it is difficult to refer these results to previous data. However, it has been confirmed that increased iodine accumulation significantly affects the antioxidant system in lettuce [46,62], radish seedlings [63], tomatoes [55], and basil [24]. Some studies have revealed the role of iodine in alleviating the negative effects of abiotic stresses, such as salinity [64,65]. Furthermore, iodine itself has been proposed as a primary inorganic antioxidant compound in marine algae [66]. However, the exact mechanisms underlying such observations still need to be discovered [9]. Data on the effect of bromine on the antioxidant capacity of plant extracts is scarce. It has only been demonstrated that application of bromine to soil (in the form of KBrO_3_) increases the content of H_2_O_2_ as well as the activity of catalase (CAT) and ascorbate peroxidase, which are important antioxidant enzymes, in carrot leaves [67]. Similar results regarding the activity of CAT and superoxide dismutase were noted in onion bulbs [68].

## 4. Materials and Methods

### 4.1. Plant Material and Treatments

A pot experiment involving the spring cultivation of the common dandelion (*Taraxacum officinale* F.H. Wiggers coll.) was conducted in a foil tunnel located on the campus of the University of Agriculture in Kraków, Poland (50°05′04.1″ N 19°57′02.1″ E). The experiment was repeated twice (two vegetation cycles in subsequent years).

The dandelion (*Taraxacum officinale* F.H. Wiggers coll.) seeds were obtained from a local producer (HerbFarm Edwin Ledczuk, Jabłoń, Poland). In both years of the study, the seeds were sown in early March into 112-cell propagation trays with 32 × 32 × 40 mm sized cells filled with peat substrate mixed with sand (2:1 *v*/*v*). The main physicochemical characteristics of the substrate were determined according to the methods described by Smoleń et al. [69] and are presented in Table S1. The plants were then transplanted into 1.5 L pots (one plant per pot) at the 3–4 leaf stage (the end of April). The pots were filled with the same substrate that was used for seed germination. The experiment included manual fertigation with the solutions of iodine (KIO_3_; Avantor Performance Materials Poland S.A., Gliwice, Poland) and bromine (KBr; Avantor Performance Materials Poland S.A., Gliwice, Poland) in the following treatments: (1) Control, (2) 10 µM KIO_3_ (10 µM I), (3) 50 µM KIO_3_ (50 µM I), (4) 10 µM KBr (10 µM Br), (5) 50 µM KBr (50 µM Br), (6) 10 µM KIO_3_ + 10 µM KBr (10 µM I + 10 µM Br), (7) 50 µM KIO_3_ + 50 µM KBr (50 µM I + 50 µM Br). The iodine doses were chosen based on our previous studies on iodine biofortification of various crop species (including dandelion) in pot experiments [55,70,71]. The selection criteria included efficient uptake of iodine and a lack of visual symptoms of its toxicity to plants. The bromine level was adjusted in order to maintain a 1:1 molar concentration ratio with iodine. The experimental setup included four replications per combination with five plants per replication (twenty plants per combination). A total of 140 plants were grown in each year of the experiment.

Iodine and bromine solutions were prepared using tap water and applied at a rate of 100 mL per pot (plant). In total, eight applications of KIO_3_ and KBr (or an equal volume of tap water in the control combination) were performed every five days during dandelion cultivation, with the first application conducted four weeks after planting. Between I and Br applications, the plants from all combinations were watered with tap water. The substrate used for dandelion cultivation contained 2.81 mg Br·kg^−1^ D.W. and 1.27 mg I·kg^−1^ D.W. The level of bromine was lower than average (10–40 mg kg^−1^), while that of iodine remained within the average value for medium loamy soils (0.3–10 mg kg^−1^) as presented by Kabata Pendias et al. [72]. Chemical analysis of tap water (described in more detail below) showed that it contained 76.5 µg Br·L^−1^ and 4.3 µg I·L^−1^, respectively. Therefore, the Br:I content ratio for water was 17.8:1, while for the substrate, it was 2.2:1.

At the end of June, four days after the last application of KIO_3_ and KBr, the dandelion plants were harvested when still at the rosette phase (before reaching the flowering stage). The rosettes and roots of the plants were collected separately by cutting the leaf rosette directly at the root collar. The leaves and roots were weighed separately to determine plant biomass, washed in tap water and distilled water, and then stored until further analysis.

In order to determine the dry matter content, a portion of fresh leaves and roots from each combination was dried at 105 °C for 24 h in order to determine the content of dry matter. The remaining plant material was freeze-dried for 48 h using a Christ Alpha 1-4 lyophilizer (Martin Christ Gefriertrocknungsanlagen GmbH, Osterode am Harz, Germany) with an external freeze-drying chamber (ARTVAC-Plus production chamber, Krzeszowice, Poland). The samples were then ground in a laboratory grinder using a 0.5 mm sieve (FRITSCH Pulverisette 14, Idar-Oberstein, Germany) and stored at −20 °C in tightly closed polyethylene bags until further analysis. All chemical analyses of the plant material described below were performed on freeze-dried leaf and root samples of dandelion.

### 4.2. Determination of Total Iodine and Bromine Content

The total iodine and bromine content of dandelion leaves and roots as well as of substrate was analyzed using an iCAP TQ ICP-MS + 250&1000 Gas Kits mass spectrometer (Thermo Fisher Scientific, Waltham, MA, USA) after alkaline extraction with tetramethylammonium hydroxide (TMAH; [28,73]). The 0.1 g samples of freeze-dried plant material (dandelion leaves or roots) or air-dried substrate were placed into 30 mL Falcon tubes. Then, 10 mL of double-distilled water and 1 mL of 25% TMAH were added, the solution was mixed, and it was incubated for 3 h at 90 °C. After cooling, samples were filled to 30 mL with double-distilled water, then mixed and centrifuged for 15 min at 4500 rpm. The supernatants were analyzed for iodine 127I (S-SQ-KED mode) and bromine 79Br|79Br.16O (S-TQ-O2 mode) using an ICP-MS/MS spectrometer. The same technique was used for the direct determination of iodine and bromine content of tap water.

### 4.3. Determination of Iodotyrosine, Benzoic and Salicylic Acids, and Their Iodine and Bromine Derivatives

The content of salicylic acid (SA), benzoic acid (BeA), 5-iodosalicylic acid (5-ISA), 5-bromosalicylic acid (5-BrSA), and iodotyrosine (I-Tyr) [commercial standards from Sigma-Aldrich/Merck KGaA, Darmstadt, Germany] in the leaves and roots of dandelion plants was analyzed using an LC-MS/MS system [Ultimate 3000 HPLC, (Thermo Fischer Scientific, Waltham, MA, USA) and 4500 Q-TRAP LC-MS/MS spectrometer (Sciex, Framingham, MA, USA)] according to the procedure described by Halka et al. [25]. The 100 mg samples of plant material (dandelion leaves or roots) were weighed into 7 mL polypropylene tubes and treated with 5 mL of 75% ethanol containing 5 ng mL^−1^ deuterium-labeled salicylic acid (SA-d4, Sigma-Aldrich/Merck KGaA, Darmstadt, Germany) as an internal standard. Then, the samples were vortexed and subjected to ultrasound-assisted extraction for 1 h at 50 °C. After extraction, the samples were centrifuged for 15 min at 4500 rpm. The resulting supernatant was filtered through a 0.22 µm nylon syringe filter (FilterBio^®^ NY Syringe Filter, Nantong Filter, Bio Membrane Co., Ltd., Nantong City, China) into a 1.5 mL Eppendorf tube. Then, 1 mL of filtrate was transferred into a chromatographic vial for analysis using the LC-MS/MS technique.

Chromatographic separation was carried out using a Luna 3 µm Phenyl Hexyl 100 Å column 150 mm × 3 mm ID, 3 µm with a Security Guard Cartridge Phenyl 4 × 2.0 mm ID precolumn (both Phenomenex, Torrance, CA, USA). The column temperature was set to 40 °C and the autosampler temperature was set to 10 °C. To determine all the aforementioned compounds, 0.3% formic acid in demineralized water (eluent A) and 0.3% formic acid (FA) in 100% methanol (eluent B) were used. A flow rate of 0.5 mL/min was applied throughout the gradient elution process as detailed in Table S2. The injection volume was 10 µL, with a total analysis time of 16 min. The HPLC column effluent was introduced directly into a mass spectrometer. Electrospray ionization (ESI) in negative mode was used. The capillary voltage and source temperature were set to −4500 V and 600 °C in negative mode and 5500 V and 600 °C in positive mode. The settings and parameters of the turbo spray ion source are listed in Table S3. Scheduled MRM mode was used for quantitative studies, with the transitions presented in Table S4. LC-MS/MS control and data processing were performed using The Analyst 1.7 with HotFix 3 software, and quantitative analysis was performed using MultiQuant 3.0.3 (version 3.0.31721.0).

### 4.4. Determination of Esculin, Chlorogenic Acid, Proline, and Phytohormones Using the LC-MS/MS Technique

In order to analyze the content of esculin, chlorogenic acid, proline, and selected phytohormones in dandelion plants, an LC-MS/MS system [Ultimate 3000 HPLC (Thermo Fischer Scientific, Waltham, MA, USA) and 4500 Q-TRAP LC-MS/MS spectrometer (Sciex, Framingham, MA, USA)] was used. Commercial standards of esculin, chlorogenic acid, jasmonic acid, and proline were obtained from Sigma Aldrich/Merck KGaA (Darmstadt, Germany). The standards of 3-indoleacetic acid (IAA) gibberellic acid syn. gibberellin A_3_ (GA_3_) were purchased from LGC Standards (Augsburg, Germany); of gibberellic acid A_4_ (GA_4_) from Toronto Research Chemicals Inc. (North York, ON, Canada); and of abscisic acid (ABA) from Duchefa Biochemie (Haarlem, The Netherlands). Prior to analysis, solid phase extraction (SPE) of the plant material was performed according to Yalçın et al. [74].

Leaf and root samples from dandelion plants (0.035 g DW) were weighed into 2 mL Eppendorf tubes and treated with 1.5 mL of 75% methanol containing 5 ng/mL deuterium-labeled 3-indoleacetic acid as an internal standard (IAA-d4, Toronto Research Chemicals Inc., North York, ON, Canada). The samples were then vortexed and subjected to ultrasound-assisted extraction for 10 min at 25 °C, after which they were placed in the freezer overnight (for 16 h). After thawing, the samples were vortexed and centrifuged for 5 min at 10,000 rpm; 1 mL of the resulting supernatant was collected (supernatant 1) into 1.5 mL Eppendorf tubes.

Re-extraction was performed on the plant tissue residue after centrifugation. A total of 500 µL of 75% methanol containing the relevant internal standards was added to the plant material. The samples were then placed in an ultrasonic bath for 1 h at 25 °C, after which they were vortexed and centrifuged for 5 min at 10,000 rpm. 500 µL of the resulting supernatant (supernatant 2) was collected from the Eppendorf tube and mixed with supernatant 1. The final extract, with a volume of 1.5 mL, was evaporated to dryness under argon (flow rate of 7 mL/min) at 50 °C using a Kamush LP-thermoblock (Lipopharm.pl, Gdańsk, Poland). The residues were redissolved in 1 mL of 20% methanol containing 0.1% formic acid, and solid-phase extraction (SPE) was performed using the SPE Strata™-X 33 µm Polymeric Reversed Phase 60 mg/3 mL ID columns (Phenomenex, Torrance, CA, USA). Next, 1 mL of the extract was transferred into a chromatographic vial for analysis by the LC-MS/MS technique. The column, precolumn, and mobile phases; chromatographic elution program; parameters of the turbo spray ion source; and data processing software were the same as those described in the above Section 4.3. The contents of GA_3_, GA_4_, JA, ABA, esculin, and chlorogenic acid were determined in negative ion mode, while those of IAA and proline were determined using positive ion mode. Electrospray ionization (ESI) in positive and negative ion modes was used. Scheduled MRM mode was used for quantitative studies, with the transitions of the individual compounds shown in Table S4 being applied.

### 4.5. Determination of the Content of Phenolic Compounds and Antioxidant Capacity

The content of phenolic compounds and the antioxidant potential of dandelion leaves and roots were determined in 70% ethanolic plant extracts. The content of phenolic compounds in dandelion was analyzed following the reaction with Folin-Ciocalteau reagent [75]. The ethanolic leaf or root extract (0.25 mL) was mixed with 0.25 mL of 25% Na_2_CO_3_, 0.125 mL of the Folin–Ciocalteau reagent (Sigma-Aldrich/Merck KGaA, Darmstadt, Germany, diluted twice with distilled water prior to the analysis), and 2.25 mL of water and mixed. After 15 min incubation in a dark place, the absorbance was measured at 760 nm (JASCO V-530 UV/Vis spectrophotometer, JASCO Corp., Tokyo, Japan). The final results were expressed as mg of chlorogenic acid (Sigma-Aldrich/Merck KGaA, Darmstadt, Germany) per g dry weight (chlorogenic acid equivalents, mg CAE·g^−1^).

The radical scavenging activity (RSA) of dandelion leaves and roots was measured using a colorimetric method that monitors the reduction in the stable synthetic free radical DPPH (2,2-diphenyl-1-picrylhydrazyl, [76]) at 517 nm using Trolox (6-hydroxy-2,5,7,8-tetramethylchroman-2-carboxylic acid; Sigma-Aldrich/Merck KGaA, Darmstadt, Germany) as an antioxidant standard. The volume of 2.8 mL of 0.1 mM DPPH (Sigma-Aldrich/Merck KGaA, Darmstadt, Germany) solution in 96% ethanol was mixed with 0.2 mL of the plant ethanolic extract. The DPPH absorbance was measured initially and after 5 min. The RSC results were expressed as μmol Trolox per 1 g of dry weight (Trolox equivalents, μmol TE·g^−1^).

The antioxidant capacity of dandelion leaves and roots was analyzed by CUPRAC (cupric ion reducing antioxidant capacity, [77]) and FRAP (ferric reducing antioxidant power, [78]) assays with Trolox as an antioxidant standard. The CUPRAC method is based on measuring copper (II)-neocuproine reduction by the antioxidants present in the analyzed sample. The volumes of 1 mL of 10 mM CuCl_2_, 1 mL of 7.5 mM neocuproine (Sigma-Aldrich/Merck KGaA, Darmstadt, Germany) in 96% ethanol, and 1 mL of 1 M NH_4_Ac buffer, pH 7.0, were mixed with 0.3 mL of the ethanolic plant extract and 0.8 mL of water. The samples were then incubated for 30 min at room temperature, and the absorbance was read at 450 nm. The results were expressed as μmol Trolox per 1 g of dry weight (Trolox equivalents, μmol TE·g^−1^).

The FRAP method measures the rate of the reduction in ferric–tripyridyl-s-triazine (Fe^+3^–TPTZ) complex by the antioxidants present in the sample [78]. Prior to the analysis, a FRAP working solution was prepared by mixing 300 mM acetate buffer solution (pH 3.6), 10 mM TPTZ (Sigma-Aldrich/Merck KGaA, Darmstadt, Germany) in 96% ethanol, and 20 mM FeCl_3_ (10:1:1, *v*:*v*:*v*). Then, 3 mL of the FRAP working solution were mixed with 0.1 mL of the plant ethanolic extract and 0.3 mL of water; samples were then incubated for 30 min at room temperature. The absorbance was then measured at 595 nm. The results were expressed as μmol Trolox per g of dry weight (Trolox equivalents, μmol TE·g^−1^).

### 4.6. Statistical Analyses

The obtained data were statistically verified using the ANOVA module of Statistica 13.3 software (StatSoft Inc., Tulsa, OK, USA) at a significance level of *p* < 0.05. Homogenous groups were identified based on a post hoc Tukey HSD test.

## 5. Conclusions

Although the direct consumption of dandelion plants, a common and widely grown medicinal plant, is not particularly popular, the increase in iodine content may provide an additional input to its well-recognized health-promoting properties. This is particularly important, as in one of the case studies, the alternative treatment for hyperthyroidism was based, among others, on Taraxaf^®^, an herbal combination containing dandelion roots [79]. Due to the significant distribution of bromine (Br) in the environment (much wider than that of iodine) and the possible environmental pollution by the residues of flame retardants and soil fumigants, dandelion plants grown in various habitats may be characterized by various levels of I and Br and, consequently, different Br:I ratios. Therefore, the quality and possible effects of such herbal material on consumers must be monitored. No distinctive symptoms of additional toxicity of Br were observed in iodine-enriched plants even though the level of Br in the leaves and roots significantly exceeded that of iodine. The physiological and biochemical responses of dandelion plants to I and Br included an increase in plant biomass as well as in the antioxidant capacity of roots and leaves. This was partially accompanied by an increase in the level of phenolic compounds. Exogenous iodine and bromine were found to slightly increase the proline content in leaves as well as the SA, BeA, and 5-BrSA content in dandelion roots. The levels of selected phytohormones were also modified by I and Br, which partially translated into increased plant biomass. Further studies on the iodine biofortification of dandelions, including the concomitant increase in Br levels, should be conducted, including the potential risk of exceeding the recommended daily intake of this element. The modifications of possible anti-cancer properties of iodine-enriched plants under the influence of Br could also be of great interest. Studies by Sularz et al. [80] have revealed that iodine-biofortified lettuce, which belongs to the Asteraceae family along with dandelions, exhibits strong antiproliferative and proapoptotic activity towards human gastrointestinal and colon cancer cell lines.

## Figures and Tables

**Table 1:** Biomass of roots, leaves, and whole dandelion plants (leaves + roots), as well as dry matter content in roots and leaves.

Treatment	Biomass			Dry Matter	
	Roots (g·plant^−1^)	Leaves (g·plant^−1^)	Whole Plant (g)	Roots (%)	Leaves (%)
Control	32.8 ± 2.13 a	32.4 ± 5.37 a	65.2 ± 3.70 a	15.4 ± 2.35 a	12.2 ± 0.74 a
10 µM I	37.5 ± 4.37 b	34.3 ± 5.97 a	71.8 ± 2.25 b	16.0 ± 2.58 a	11.8 ± 0.49 a
50 µM I	37.8 ± 3.27 b	32.8 ± 5.30 a	70.6 ± 2.14 b	16.5 ± 2.57 a	11.6 ± 0.57 a
10 µM Br	38.5 ± 4.91 b	32.1 ± 5.68 a	70.6 ± 1.39 b	14.6 ± 1.74 a	11.2 ± 0.26 a
50 µM Br	41.4 ± 6.10 bc	34.3 ± 6.82 a	75.7 ± 1.88 b	15.8 ± 2.78 a	12.1 ± 0.53 a
10 µM I + 10 µM Br	40.9 ± 4.43 bc	36.3 ± 6.83 a	77.2 ± 2.96 bc	14.3 ± 2.54 a	12.1 ± 0.73 a
50 µM I + 50 µM Br	45.0 ± 8.35 c	39.0 ± 6.25 a	84.0 ± 2.85 c	17.2 ± 1.67 a	11.2 ± 0.32 a

Means (±SD) followed by different letters separately for leaves and roots differ significantly at *p* < 0.05. Means from two cycles of dandelion plant cultivation; n = 8.

**Table 2:** Concentrations of iodine and bromine as well as the Br:I quantitative ratio in the roots and leaves of dandelion plants.

Part of Plant	Treatment	Total Content		Quantitative Ratio Br:I
		mg I·kg^−1^ DW	mg Br·kg^−1^ DW	
Leaves	Control	0.34 ± 0.009 a	9.04 ± 1.677 a	26.6:1
	10 µM I	4.32 ± 0.810 b	8.36 ± 0.872 a	1.9:1
	50 µM I	26.63 ± 8.702 c	11.08 ± 1.227 a	0.4:1
	10 µM Br	0.44 ± 0.033 a	31.08 ± 0.531 b	70.6:1
	50 µM Br	0.27 ± 0.016 a	156.46 ± 11.062 d	579.5:1
	10 µM I + 10 µM Br	3.80 ± 0.969 b	52.22 ± 4.975 c	13.7:1
	50 µM I + 50 µM Br	30.39 ± 5.191 d	241.01 ± 49.417 e	7.9:1
Roots	Control	0.29 ± 0.050 a	4.06 ± 0.443 a	14.0:1
	10 µM I	1.20 ± 0.201 b	4.38 ± 0.048 a	3.6:1
	50 µM I	5.81 ± 1.250 d	5.40 ± 0.217 a	0.9:1
	10 µM Br	0.35 ± 0.018 a	11.59 ± 1.286 c	33.1:1
	50 µM Br	0.33 ± 0.019 a	41.42 ± 5.779 d	125.5:1
	10 µM I + 10 µM Br	1.03 ± 0.153 b	12.21 ± 0.880 c	11.8:1
	50 µM I + 50 µM Br	5.11 ± 1.184 c	38.12 ± 5.358 d	7.5:1

Means (±SD) followed by different letters separately for leaves and roots differ significantly at *p* < 0.05. The results of I and Br total content and the Br:I quantitative ratio are means from two cycles of dandelion plant cultivation; n = 8.

**Table 3:** Concentrations of esculin, chlorogenic acid, and total phenolic compounds in leaves and roots of dandelion plants.

Part of Plant	Treatment	Esculin (mg·kg^−1^ DW)	Chlorogenic Acid (mg·kg^−1^ DW)	Total Phenols (g·kg^−1^ DW)
		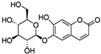	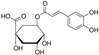	
Leaves	Control	34.7 ± 1.35 a	365.0 ± 14.16 ab	42.8 ± 0.96 bc
	10 µM I	26.5 ± 0.83 a	387.8 ± 10.26 b	42.6 ± 1.08 c
	50 µM I	30.6 ± 2.37 a	399.5 ± 4.62 c	51.3 ± 2.23 d
	10 µM Br	28.0 ± 2.67 a	305.5 ± 9.92 a	39.3 ± 0.90 a
	50 µM Br	29.8 ± 1.48 a	354.6 ± 13.44 ab	40.6 ± 1.84 ab
	10 µM I + 10 µM Br	24.3 ± 0.76 a	324.3 ± 16.15 ab	41.6 ± 0.24 bc
	50 µM I + 50 µM Br	28.8 ± 1.65 a	393.3 ± 15.17 bc	41.0 ± 0.48 ab
Roots	Control	0.082 ± 0.005 a	186.4 ± 3.65 a	8.1 ± 0.20 ab
	10 µM I	0.104 ± 0.005 a	203.4 ± 4.49 abc	8.0 ± 0.32 a
	50 µM I	0.104 ± 0.007 a	222.6 ± 6.12 c	8.7 ± 0.38 ab
	10 µM Br	0.104 ± 0.007 a	266.0 ± 5.01 d	11.3 ± 0.41 d
	50 µM Br	0.111 ± 0.003 a	213.2 ± 4.17 bc	9.5 ± 0.22 c
	10 µM I + 10 µM Br	0.104 ± 0.001 a	189.0 ± 4.15 ab	8.6 ± 0.45 ab
	50 µM I + 50 µM Br	0.096 ± 0.004 a	194.3 ± 1.26 ab	8.4 ± 0.33 ab

Means (±SD) followed by different letters separately for leaves and roots differ significantly at *p* < 0.05. Means from two cycles of dandelion plant cultivation; n = 8.

**Table 4:** Antioxidant capacity of the extracts of leaves and roots of dandelion plants.

Part of Plant	Treatments	Antioxidant Capacity (µmol TE·g^−1^ DW)		
		CUPRAC	FRAP	DPPH
Leaves	Control	186.1 ± 5.31 ab	151.6 ± 6.50 a	68.8 ± 4.18 ab
	10 µM I	202.4 ± 12.19 bc	152.9 ± 4.30 a	72.2 ± 2.63 b
	50 µM I	204.8 ± 8.19 c	167.4 ± 6.17 cd	73.6 ± 3.76 b
	10 µM Br	188.1 ± 6.45 ab	162.4 ± 4.65 bc	67.8 ± 3.56 ab
	50 µM Br	184.3 ± 12.00 a	155.5 ± 5.29 ab	64.7 ± 2.49 ab
	10 µM I + 10 µM Br	210.9 ± 10.32 c	178.1 ± 6.07 e	65.2 ± 6.62 ab
	50 µM I + 50 µM Br	199.4 ± 9.37 bc	171.2 ± 1.11 de	62.4 ± 8.62 a
Roots	Control	67.8 ± 6.12 ab	24.5 ± 0.53 a	24.5 ± 1.38 a
	10 µM I	65.7 ± 3.54 a	23.6 ± 0.89 a	26.2 ± 1.65 b
	50 µM I	75.3 ± 2.89 cd	25.7 ± 0.58 b	27.5 ± 1.23 bc
	10 µM Br	94.7 ± 2.97 e	33.2 ± 1.50 d	32.6 ± 0.49 d
	50 µM Br	80.7 ± 1.82 d	29.3 ± 1.02 c	29.5 ± 0.93 c
	10 µM I + 10 µM Br	73.3 ± 4.02 bc	26.6 ± 0.97 b	25.2 ± 1.32 ab
	50 µM I + 50 µM Br	72.0 ± 2.39 abc	25.3 ± 1.21 ab	25.6 ± 0.99 ab

Means (±SD) followed by different letters separately for leaves and roots differ significantly at *p* < 0.05. Means from two cycles of dandelion plant cultivation; n = 8.

**Table 5:** Concentrations of phytohormones IAA, GA_3_, GA_4_, JA, and ABA, as well as of proline, in dandelion leaves and roots.

Part of Plant	Treatment	IAA	GA_3_	GA_4_	JA	ABA	Proline
				(µg·kg^−1^ DW)			(mg·kg^−1^ DW)
Leaves	Control	599.3 ± 24.08 b	41.9 ± 1.41 bc	283.4 ± 18.26 a	412.2 ± 12.22 e	421.8 ± 10.17 bc	8.59 ± 0.056 a
	10 µM I	548.1 ± 52.17 b	133.5 ± 12.05 d	250.2 ± 12.07 a	347.3 ± 4.35 d	551.2 ± 20.71 d	12.31 ± 1.036 b
	50 µM I	645.6 ± 26.72 b	23.9 ± 1.21 ab	261.1 ± 13.37 a	166.2 ± 2.29 a	455.6 ± 18.07 c	11.01 ± 0.926 b
	10 µM Br	602.8 ± 31.02 b	9.9 ± 0.26 a	315.7 ± 30.09 a	284.4 ± 3.67 c	363.2 ± 4.06 ab	10.54 ± 0.569 ab
	50 µM Br	568.3 ± 40.57 b	25.7 ± 2.31 ab	270.3 ± 16.21 a	145.1 ± 6.09 a	392.2 ± 16.43 bc	11.94 ± 0.700 b
	10 µM I + 10 µM Br	487.7 ± 29.26 b	159.5 ± 1.20 d	410.8 ± 7.77 b	201.3 ± 3.26 b	300.8 ± 7.63 a	12.29 ± 1.124 b
	50 µM I + 50 µM Br	281.5 ± 6.55 a	71.8 ± 6.90 c	273.3 ± 9.70 a	261.7 ± 5.49 c	392.2 ± 21.05 bc	11.16 ± 0.761 b
Roots	Control	72.49 ± 1.20 a	311.8 ± 19.98 ab	446.7 ± 7.91 a	285.9 ± 19.96 a	45.1 ± 5.43 bc	10.84 ± 0.276 b
	10 µM I	149.2 ± 15.72 b	386.9 ± 6.91 ab	569.6 ± 63.93 a	525.8 ± 21.93 ab	29.6 ± 2.51 ab	8.15 ± 0.249 a
	50 µM I	282.2 ± 36.03 bc	363.4 ± 30.67 ab	484.4 ± 13.69 a	946.2 ± 76.13 c	33.7 ± 3.11 abc	7.59 ± 0.368 a
	10 µM Br	239.4 ± 13.34 bc	416.9 ± 24.22 b	594.3 ± 7.08 a	673.0 ± 41.23 b	51.0 ± 6.43 c	10.40 ± 0.650 b
	50 µM Br	329.2 ± 44.82 c	316.9 ± 26.51 ab	507.6 ± 34.26 a	660.1 ± 48.64 b	29.1 ± 0.21 ab	10.37 ± 0.420 b
	10 µM I + 10 µM Br	403.1 ± 51.73 c	371.1 ± 2.75 ab	520.5 ± 3.40 a	995.3 ± 69.71 c	52.4 ± 0.13 c	10.24 ± 0.514 b
	50 µM I + 50 µM Br	281.0 ± 23.97 bc	288.2 ± 16.91 a	551.3 ± 20.10 a	595.7 ± 7.45 b	19.4 ± 1.81 a	10.69 ± 0.586 b

Means (±SD) followed by different letters separately for leaves and roots differ significantly at *p* < 0.05. Means from two cycles of dandelion plant cultivation; n = 8.

## Data Availability

Dataset available on request from the authors.

## Supplementary Materials

The following supporting information can be downloaded at https://www.mdpi.com/article/10.3390/molecules30102239/s1, Graphical summary of the studies. Table S1. Main physicochemical characteristics of the substrate used for plant propagation and cultivation in a pot experiment; Table S2. Chromatographic gradient: A—0.3% formic acid in H_2_O; B—0.3% formic acid in methanol; Table S3. Parameters of the turbo spray ion source; Table S4. Precursor/product transitions of the analytes iodotyrosine, esculin, chlorogenic acid, benzoic acid, salicylic acid, 5-iodosalicylic acid, and 5-bromosalicylic acid and the phytohormones NAA, IAA, GA_3_, GA_4_, JA, and ABA.
