# Supplementary material for: The Effect of Bromine and Iodine on the Plant Growth, Phytochemical Composition and Antioxidant Capacity of Dandelion (Taraxacum officinale F.H. Wiggers Coll.) Plants
Source: Molecules. 2025 May 21;30(10):2239. doi: 10.3390/molecules30102239 (PMC12114093; doi:10.3390/molecules30102239)
Supplement: Supplementary file 1 [file molecules-30-02239-s001.zip › molecules-3566896-supplementary.docx]

Supporting information file

**The effect of bromine and iodine on plant growth, phytochemical composition and antioxidant capacity of dandelion (*****Taraxacum officinale*** **F.H. Wiggers coll.) plants**

Sylwester Smoleń^a^, Iwona Ledwożyw-Smoleń^a^, Marta Liszka-Skoczylas^b^, Joanna Pitala^c^, Łukasz Skoczylas^d^

a - Department of Plant Biology and Biotechnology, Faculty of Biotechnology and Horticulture, University of Agriculture in Krakow, al. Mickiewicza 21, 31-120 Krakow, Poland

b - Department of Engineering and Machinery for Food Industry, Faculty of Food Technology, University of Agriculture in Krakow, al. Mickiewicza 21, 31-120 Krakow, Poland

c - Laboratory of Mass Spectrometry, Faculty of Biotechnology and Horticulture, University of Agriculture in Krakow, al. Mickiewicza 21, 31-120 Krakow, Poland

d - Department of Plant Product Technology and Nutrition Hygiene, Faculty of Food Technology, University of Agriculture in Krakow, al. Mickiewicza 21, 31-120 Krakow, Poland

**Table S1.** Main physicochemical characteristics of the substrate used for plant propagation and cultivation in a pot experiment

| pH | EC | Eh | N-NH_4_^+^ | N-NO_3_^-^ | Ca | K | Mg | Na | P | S | Cl^-^ | | | I | Br |
| --- | --- | --- | --- | --- | --- | --- | --- | --- | --- | --- | --- | --- | --- | --- | --- |
|  | mS/cm | mV | mg·L | | | | | | | | | | | | |
| 4.01 | 0.26 | 383.8 | 96.75 | 83.7 | 1913.3 | 196.2 | 72.6 | 20.9 | 12.3 | 1705.3 | | 13.59 | 1.27 | | 2.81 |

The chemical parameters of the substrate were determined according to methods described by Smoleń et al. (2021). Iodine and bromine were analyzed after extraction in TMAH (tetramethylammonium hydroxide) according to the procedure for iodine by Smoleń et al. (2021).

Smoleń, S., Czernicka, M., Kowalska, I., Kȩska, K., Halka, M., Grzebelus, D., ... & Kováčik, P. (2021). New aspects of uptake and metabolism of non-organic and organic iodine compounds—the role of vanadium and plant-derived thyroid hormone analogs in lettuce. Frontiers in Plant Science, 12, 653168. Doi: [10.3389/fpls.2021.653168](https://doi.org/10.3389/fpls.2021.653168)

**Table S2.** Chromatographic gradient: A – 0.3% formic acid in H_2_O; B – 0.3% formic acid in methanol.

| nr | Retention time, min | %A | %B |
| --- | --- | --- | --- |
| 1. | 0.0 | 60 | 40 |
| 2. | 2.0 | 60 | 40 |
| 3. | 5.0 | 30 | 70 |
| 4. | 8.0 | 2 | 98 |
| 5. | 13.5 | 2 | 98 |
| 6. | 13.6 | 60 | 40 |
| 7. | 16.0 | 60 | 40 |

**Table S3.** Parameters of Turbo Spray Ion Source

| Parameter | Negative ion mode | Positive ion mode |
| --- | --- | --- |
| IonSpray Voltage | -4500 V | 5500V |
| Temperature | 600°C | 600°C |
| Curtain gas (CUR) | 30 | 30 |
| Collision Gas | medium | medium |
| Ion source gas (GS1) | 50 | 50 |
| Ion source gas (GS2) | 65 | 65 |

**Table S4.** Precursor/product transitions of analytes: iodotyrosine, esculin, chlorogenic acid, benzoic acid, salicylic acid, 5-iodosalicylic acid, 5-bromosalicylic acid and phytohormones: NAA, IAA, GA_3_, GA_4_, JA and ABA

| **Analyte** | **Precursor ion (m/z)** | **Product ion (m/z)** | **transition** |
| --- | --- | --- | --- |
| **Data for subchapter:** **“Determination of iodotyrosine, benzoic and salicylic acids and their iodine and bromine derivatives”** | | | |
| Salicylic acid (SA) | 136.8 | 93.1 | quantifying |
| Salicylic acid (SA) | 136.8 | 64.9 | qualifying |
| Benzoic acid (BeA) | 120.9 | 76.9 | quantifying |
| Benzoic acid (BeA) | 120.9 | 92.8 | qualifying |
| Iodotyrosine (I-Tyr) | 306.1 | 126.8 | quantifying |
| Iodotyrosine (I-Tyr) | 306.1 | 288.8 | qualifying |
| 5-iodosalicylic acid (5-SA) | 262.9 | 126.7 | quantifying |
| 5-iodosalicylic acid (5-SA) | 262.9 | 218.8 | qualifying |
| 5-bromosalicylic acid (5-BrSA) | 216.4 | 172.7 | quantifying |
| 5-bromosalicylic acid (5-BrSA) | 216.4 | 80.8 | qualifying |
| **Data for subchapter: “Determination of esculin, chlorogenic acid, proline  and phytohormones by LC-MS/MS technique”** | | | |
| Esculin | 339.1 | 176.8 | quantifying |
| Esculin | 339.1 | 176.8 | qualifying |
| Chlorogenic acid | 353.2 | 190.6 | quantifying |
| Chlorogenic acid | 353.2 | 84.8 | qualifying |
| Proline | 115.9 | 69.9 | quantifying |
| Proline | 116.9 | 69.9 | qualifying |
| Jasmonic acid (JA) | 209.15 | 58.77 | quantifying |
| Jasmonic acid (JA) | 209.15 | 165.0 | qualifying |
| 3-indoleacetic acid (IAA) | 176.1 | 130.1 | quantifying |
| 3-indoleacetic acid (IAA) | 176.1 | 103.0 | qualifying |
| Gibberellin A_3_ (GA_3_) | 345.0 | 142.8 | quantifying |
| Gibberellin A_3_ (GA_3_) | 345.0 | 238.9 | qualifying |
| Gibberellic acid A_4_ (GA_4_) | 331.0 | 287.0 | quantifying |
| Gibberellic acid A_4_ (GA_4_) | 331.0 | 257.0 | qualifying |
| Abscisic acid (ABA) | 263.03 | 152.94 | quantifying |
| Abscisic acid (ABA) | 263.03 | 219.0 | qualifying |
