# Supplementary figures and images for: The Effect of Bromine and Iodine on the Plant Growth, Phytochemical Composition and Antioxidant Capacity of Dandelion (Taraxacum officinale F.H. Wiggers Coll.) Plants
Source: Molecules. 2025 May 21;30(10):2239. doi: 10.3390/molecules30102239 (PMC12114093; doi:10.3390/molecules30102239)

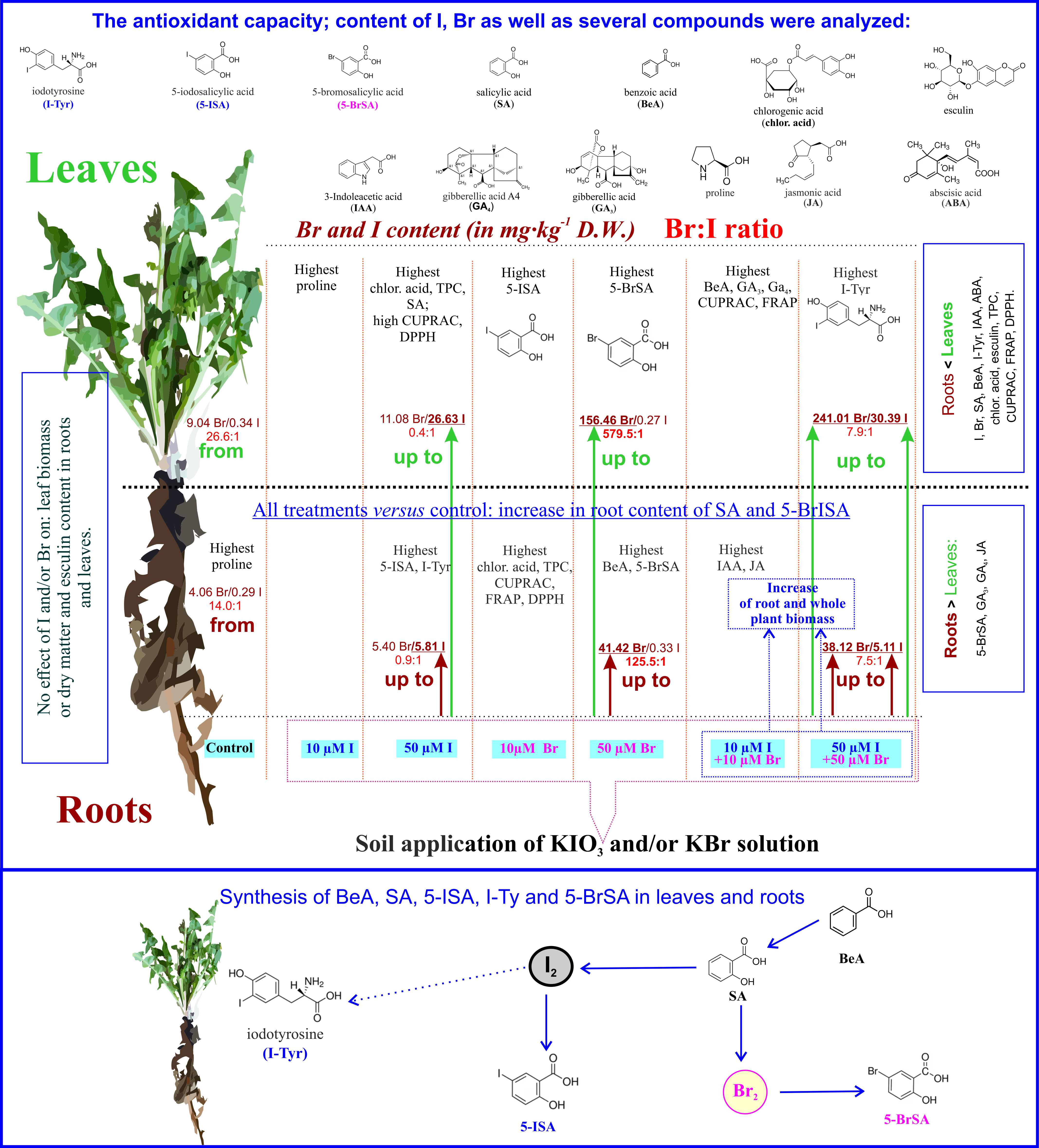

Supplement: Supplementary file 1 [file molecules-30-02239-s001.zip › Dandelion I and Br 2024 summary final.tif]
